# Persistent Parvovirus B19 Viremia Despite Repeated Intravenous Immunoglobulin Therapy After Kidney Transplantation: A Case Report

**DOI:** 10.7759/cureus.111825

**Published:** 2026-06-30

**Authors:** Mohammed Hamzaoui, Imane Machmachi, Chaimae Boullit, Intissar Haddiya, Yassamine Bentata

**Affiliations:** 1 Nephrology, Dialysis and Kidney Transplantation Unit, Centre Hospitalo-Universitaire Mohammed VI d'Oujda, Oujda, MAR; 2 Laboratory of Epidemiology, Clinical Research and Public Health, Faculty of Medicine, Mohamed I University, Oujda, MAR

**Keywords:** anemia, donor-specific antibodies, intravenous immunoglobulin, kidney transplantation, parvovirus b19, persistent viremia

## Abstract

Parvovirus B19 infection is an increasingly recognized opportunistic infection in kidney transplant recipients and should be considered in patients presenting with unexplained anemia after transplantation.

We report the case of a 31-year-old kidney transplant recipient who developed Parvovirus B19 infection 27 days after transplantation. The patient presented with a flu-like syndrome, severe leukopenia, and erythropoietin-resistant anemia. Quantitative polymerase chain reaction revealed a viral load exceeding 10⁹ copies/mL. Intravenous immunoglobulin therapy was administered, resulting in hematological improvement. However, despite three courses of intravenous immunoglobulin therapy and prolonged reduction of immunosuppressive treatment, persistent Parvovirus B19 viremia was documented for approximately 40 months. During follow-up, donor-specific antibodies developed, and the kidney allograft biopsy demonstrated recurrent IgA nephropathy without evidence of rejection.

Viral load progressively decreased to 280 copies/mL in December 2022 before becoming undetectable in January 2023, allowing progressive reintroduction of mycophenolate mofetil.

This case highlights the challenges associated with achieving viral clearance in kidney transplant recipients with Parvovirus B19 infection and emphasizes the importance of long-term virological and immunological monitoring.

## Introduction

Viral infections remain a major cause of morbidity in kidney transplant recipients because of long-term immunosuppressive therapy. Among the most frequently encountered opportunistic viral infections are cytomegalovirus, BK polyomavirus, Epstein-Barr virus, and Parvovirus B19 [1,2]. Parvovirus B19 infection is increasingly recognized in kidney transplant recipients, with reported prevalence varying widely according to the studied population and diagnostic methods used [3,4].The infection typically occurs during the first months after transplantation and should be suspected in patients presenting with unexplained anemia, particularly when associated with a viral syndrome and resistance to erythropoietin therapy [3,5].

Parvovirus B19 infection is characterized by a broad spectrum of clinical manifestations, ranging from asymptomatic viremia to severe hematological complications, including pure red cell aplasia and profound anemia [5,6]. Diagnosis may be challenging because of the nonspecific clinical presentation and the limited sensitivity of serological tests in immunocompromised patients, making polymerase chain reaction (PCR) the diagnostic method of choice [3,7]. Treatment is mainly based on reduction of immunosuppressive therapy and administration of intravenous immunoglobulins [7,8]. We report a case of Parvovirus B19 infection occurring early after kidney transplantation, characterized by persistent viremia for 40 months despite repeated courses of intravenous immunoglobulin therapy and prolonged adjustment of immunosuppressive treatment.

This work was previously presented as a meeting abstract at the Société Francophone de Néphrologie, Dialyse et Transplantation (SFNDT) Congress 2022. The present manuscript provides substantially expanded follow-up data, including long-term virological outcome, donor-specific antibody evolution, and kidney allograft biopsy findings.

## Case presentation

A 31-year-old Moroccan man with end-stage kidney disease secondary to chronic glomerulonephritis was referred to our department. Before dialysis initiation, he presented with nephrotic-range proteinuria (10 g/24 h), microscopic hematuria, recurrent episodes of fever, and dark-colored urine. Native kidney biopsy revealed chronic glomerulonephritis with diffuse mesangial and granular C3 deposits, whereas IgA staining was unavailable at the time of diagnosis. Immunological investigations were negative. The patient subsequently underwent maintenance hemodialysis for five years.

In September 2019, he underwent living-related kidney transplantation from his paternal aunt. The donor shared a haploidentical human leukocyte antigen (HLA) class I profile and HLA-DR13. Pre-transplant anti-HLA antibodies were detected, but donor-specific antibodies (DSA) were absent. Induction immunosuppression consisted of basiliximab (20 mg on days 1 and 4). Maintenance therapy included mycophenolate mofetil (1 g twice daily), tacrolimus (5 mg twice daily, adjusted according to trough levels), and corticosteroids.

Immediate graft function was achieved, with a discharge serum creatinine of approximately 71 µmol/L (8 mg/L). Early after transplantation, the patient developed severe pulmonary sepsis complicated by acute kidney injury, with serum creatinine rising to 796 µmol/L (90 mg/L). Broad-spectrum antibiotic therapy resulted in favorable clinical and biological recovery, with subsequent improvement in kidney function.

Approximately one month after transplantation, the patient presented with a flu-like syndrome, fever (39 °C), severe leukopenia (450 cells/mm³), and progressive anemia (hemoglobin 6.7 g/dL). Cytomegalovirus infection was initially suspected, leading to a reduction in the mycophenolate mofetil dosage, discontinuation of valacyclovir, and initiation of ganciclovir therapy. However, cytomegalovirus polymerase chain reaction (PCR) testing was negative, and no clinical improvement was observed.

The patient subsequently developed erythropoietin-resistant anemia. Parvovirus B19 serological testing (IgM and IgG) was not performed because these assays were unavailable at our institution at the time of diagnosis. Quantitative PCR revealed Parvovirus B19 viremia exceeding 10⁹ copies/mL. Intravenous immunoglobulin therapy was administered at a dose of 0.4 g/kg/day for 5 consecutive days (total dose 100 g), resulting in significant hematological improvement. Transient acute kidney injury occurred after immunoglobulin administration and resolved following hydration and tacrolimus dose reduction.

Serial monitoring demonstrated a decrease in Parvovirus B19 viral load to 1,242 copies/mL, followed by a subsequent increase to 9,000 copies/mL in August 2020, prompting a second course of intravenous immunoglobulin therapy because of recurrent anemia associated with persistent viremia. A third course was administered in August 2021 because of persistent viremia and recurrent hematological deterioration. In December 2020, the patient was hospitalized for COVID-19 infection and recovered with supportive treatment. In July 2021, he was readmitted for management of an infectious syndrome.

Because of persistent viremia, mycophenolate mofetil was discontinued, tacrolimus exposure was reduced with a target trough level of 3-4 ng/mL, and corticosteroid therapy was maintained. Hematological recovery was achieved after each course of intravenous immunoglobulin therapy, whereas virological response remained incomplete. Despite three courses of intravenous immunoglobulin therapy and prolonged reduction of immunosuppressive treatment, persistent Parvovirus B19 viremia was documented for approximately 40 months. Viral load progressively decreased to 280 copies/mL in December 2022 before becoming undetectable in January 2023.

Monitoring of anti-HLA antibodies revealed the appearance of donor-specific antibodies during follow-up. In February 2020, anti-Cw06 DSA were detected with a mean fluorescence intensity (MFI) of 1,328. In January 2021, anti-DQ2 DSA appeared with an MFI of 22,227. In January 2022, anti-DQ2 antibodies persisted with an MFI of 23,337, associated with anti-B53 (MFI 369) and anti-CW4 (MFI 207) antibodies.

In January 2022, the patient was hospitalized for acute kidney injury with serum creatinine of 159 µmol/L and proteinuria of 1.3 g/24 h. Kidney allograft biopsy demonstrated diffuse mesangial IgA and C3 deposits associated with mesangial expansion, focal segmental hyalinosis involving a single glomerulus, minimal chronic tubulointerstitial changes, and mild tubular epithelial necrosis. No endocapillary or extracapillary proliferation and no histological evidence of rejection were identified. The Oxford classification score was M0E0S0T0C0. These findings were consistent with recurrent IgA nephropathy.

A nephroprotective strategy was adopted with a target tacrolimus trough concentration of 4 ng/mL. Following viral clearance, mycophenolate mofetil was progressively reintroduced while tacrolimus and corticosteroid therapy were continued.

At the latest follow-up, serum creatinine remained stable at approximately 115 µmol/L, proteinuria was negative, and anti-DQ2 donor-specific antibodies persisted with an MFI of approximately 23,000.

The main laboratory findings are summarized in Table 1. The evolution of donor-specific antibodies is summarized in Table 2, whereas the major clinical events during follow-up are presented in Table 3.

**Table 1:** Main laboratory findings during follow-up AKI: acute kidney injury; CMV: cytomegalovirus; IVIG: intravenous immunoglobulin; PCR: polymerase chain reaction

Category	Parameter	Result	Reference range
Hematology	Hemoglobin	6.7 g/dL	13–18 g/dL
	Hematocrit	18%	40–54%
	Red blood cells	2.16 ×10⁶/µL	5–5.5 ×10⁶/µL
	White blood cells	1.94 ×10³/µL	4–10 ×10³/µL
	Neutrophils	0.62 ×10³/µL	1.5–7 ×10³/µL
	Lymphocytes	0.93 ×10³/µL	1–4 ×10³/µL
	Platelets	223 ×10³/µL	150–400 ×10³/µL
Renal function	Baseline serum creatinine after transplantation	71 µmol/L	64–111 µmol/L
	Peak creatinine during septic AKI	796 µmol/L	64–111 µmol/L
	Creatinine at recurrent IgAN	159 µmol/L (18 mg/L)	64–111 µmol/L
	Proteinuria at recurrence	1.3 g/24 h	<0.15 g/24 h
	Final serum creatinine	115 µmol/L	64–111 µmol/L
Virology	CMV PCR	Negative	Not detected
	Initial Parvovirus B19 PCR	>10⁹ copies/mL	<250 copies/mL
	Post-IVIG viral load	1,242 copies/mL	<250 copies/mL
	Viral relapse	9,000 copies/mL	<250 copies/mL
	Pre-clearance viral load	280 copies/mL	<250 copies/mL
	Final PCR	Negative (<250 copies/mL)	<250 copies/mL
Immunology	Pre-transplant DSA	Absent	Absent
	Anti-Cw06 DSA	1,328 MFI	Absent
	Anti-DQ2 DSA	22,227 MFI	Absent
	Persistent anti-DQ2 DSA	23,337 MFI	Absent
	Anti-B53 antibody	369 MFI	Absent
	Anti-CW4 antibody	207 MFI	Absent

**Table 2:** Evolution of anti-HLA antibodies and donor-specific antibodies during follow-up DSA: donor-specific antibodies; MFI: mean fluorescence intensity; HLA: human leukocyte antigen

Date	HLA antibodies	DSA	MFI
February 2020	Class I positive	Anti-Cw06	1328
January 2021	Class II positive	Anti-DQ2	22227
January 2022	Class I and II positive	Anti-DQ2	23337
January 2022	Class I and II positive	Anti-B53	369
January 2022	Class I and II positive	Anti-Cw4	207

**Table 3:** Major clinical events during follow-up

Time	Event
September 2019	Kidney transplantation
October 2019	Parvovirus B19 infection (PCR >10⁹ copies/mL)
December 2019	First IVIG course
August 2020	PCR relapse (9000 copies/mL), second IVIG course
December 2020	COVID-19 infection
January 2021	Anti-DQ2 DSA detected
August 2021	Third IVIG course
January 2022	Graft biopsy: recurrent IgA nephropathy (Oxford score M0E0S0T0C0)
December 2022	PCR 280 copies/mL
January 2023	PCR negative

## Discussion

Parvovirus B19 infection is a recognized cause of severe anemia in kidney transplant recipients and typically occurs during the first months after transplantation, when immunosuppressive therapy is most intense [3,5]. In the review by Eid et al., most cases occurred during the first months after transplantation, with a median time to diagnosis of six weeks [5]. In our patient, infection developed 27 days after transplantation, which is consistent with previously reported observations.

The diagnosis of Parvovirus B19 infection remains challenging because of its nonspecific clinical presentation. Fever, flu-like symptoms, leukopenia, and progressive anemia may mimic cytomegalovirus infection, drug toxicity, or other opportunistic infections commonly encountered after kidney transplantation [3,5]. In our patient, cytomegalovirus (CMV) infection was initially suspected despite a negative CMV PCR result. The subsequent development of erythropoietin-resistant anemia prompted further investigations and ultimately led to the diagnosis of Parvovirus B19 infection. This observation emphasizes the importance of considering Parvovirus B19 infection in kidney transplant recipients presenting with severe anemia associated with leukopenia, particularly when routine investigations remain inconclusive.

In immunocompromised patients, serological assays may have limited diagnostic value because of impaired humoral immune responses. Consequently, quantitative PCR remains the preferred diagnostic tool and is essential for both diagnosis and follow-up [3,7]. Parvovirus B19 serological testing (IgM and IgG) was not performed in our patient because these assays were unavailable at our institution at the time of diagnosis. Our patient presented with a viral load exceeding 10⁹ copies/mL, a finding frequently reported in delayed or severe infections and reflecting uncontrolled viral replication [3,9].

Currently, no specific antiviral therapy is available for Parvovirus B19 infection. Reduction of immunosuppressive therapy and administration of intravenous immunoglobulins remain the cornerstone of treatment [7,8]. Most published cases report rapid hematological improvement after one or two courses of intravenous immunoglobulin therapy, whereas viral clearance usually occurs within several weeks or months [5,8]. In our patient, repeated courses of intravenous immunoglobulin therapy were administered because of recurrent erythropoietin-resistant anemia associated with persistent high-level viremia. In contrast, our patient exhibited persistent viremia despite three courses of intravenous immunoglobulin therapy, withdrawal of mycophenolate mofetil, and reduction of tacrolimus exposure. Although anemia improved after treatment, viral replication persisted for approximately 40 months before eventual clearance. This prolonged virological persistence represents the most remarkable feature of our case and highlights the difficulty of achieving complete viral eradication in certain kidney transplant recipients.

Parvovirus B19 PCR progressively decreased to 280 copies/mL in December 2022 and became undetectable in January 2023, allowing progressive reintroduction of mycophenolate mofetil. This observation illustrates that viral eradication may be achieved after several years of follow-up despite repeated relapses and prolonged adaptation of immunosuppressive therapy.

Another noteworthy aspect of our observation was the development of donor-specific antibodies during follow-up. Reduction of immunosuppressive therapy is often necessary to facilitate viral control but may increase the risk of alloimmune sensitization. In addition, chronic inflammatory stimulation related to persistent viral infection has been associated with increased production of anti-HLA antibodies [10,11]. Therefore, the emergence of donor-specific antibodies in our patient was likely multifactorial, resulting from both prolonged reduction of immunosuppressive therapy and persistent immune stimulation related to chronic Parvovirus B19 infection. Nevertheless, a direct causal relationship cannot be established.

Kidney allograft biopsy demonstrated recurrent IgA nephropathy without histological evidence of rejection. Several glomerular lesions have been reported in association with Parvovirus B19 infection, including focal segmental glomerulosclerosis, collapsing glomerulopathy, endocapillary proliferative glomerulonephritis, and thrombotic microangiopathy [12,13]. However, the histological findings observed in our patient were more consistent with recurrence of the native kidney disease than with direct Parvovirus B19-related renal injury.

This report has several limitations. As a single-case report, the findings cannot be generalized to all kidney transplant recipients with Parvovirus B19 infection. In addition, the absence of Parvovirus B19 serological testing limited complete virological characterization. Nevertheless, the exceptionally prolonged duration of persistent viremia with eventual viral clearance after long-term follow-up provides valuable clinical insight into the management of this uncommon condition.

## Conclusions

Persistent Parvovirus B19 infection remains an uncommon but important cause of erythropoietin-resistant anemia after kidney transplantation. This case illustrates that viral clearance may be difficult to achieve despite repeated courses of intravenous immunoglobulin therapy and prolonged adaptation of immunosuppressive treatment.

Long-term monitoring of viral load, donor-specific antibodies, and graft function is essential in affected patients. Our observation demonstrates that complete viral clearance may ultimately be achieved after prolonged follow-up despite repeated relapses and persistent viremia. Although this report describes a single patient and its findings cannot be generalized, it highlights important clinical challenges in the diagnosis and long-term management of persistent Parvovirus B19 infection in kidney transplant recipients.

## References

[1] Alangaden GJ, Thyagarajan R, Gruber SA (2006). Infectious complications after kidney transplantation: current epidemiology and associated risk factors. Clin Transplant.

[2] Blazquez-Navarro A, Dang-Heine C, Wittenbrink N (2018). BKV, CMV, and EBV interactions and their effect on graft function one year post-renal transplantation: results from a large multicentre study. EBioMedicine.

[3] Bentata Y (2021). Parvovirus B19 in kidney transplantation: key points and essential pitfalls to know. Infect Dis (Lond).

[4] Ki CS, Kim IS, Kim JW (2005). Incidence and clinical significance of human parvovirus B19 infection in kidney transplant recipients. Clin Transplant.

[5] Eid AJ, Brown RA, Patel R, Razonable RR (2006). Parvovirus B19 infection after transplantation: a review of 98 cases. Clin Infect Dis.

[6] Baek CH, Kim H, Yang WS, Han DJ, Park SK (2017). Risk factors and long-term outcomes of parvovirus B19 infection in kidney transplant patients. Transpl Infect Dis.

[7] Eid AJ, Ardura MI (2019). Human parvovirus B19 in solid organ transplantation: guidelines from the American Society of Transplantation Infectious Diseases Community of Practice. Clin Transplant.

[8] Liefeldt L, Buhl M, Schweickert B (2002). Eradication of parvovirus B19 infection after renal transplantation requires reduction of immunosuppression and high-dose immunoglobulin therapy. Nephrol Dial Transplant.

[9] Park JB, Kim DJ, Woo SY (2009). Clinical implications of quantitative real time-polymerase chain reaction of parvovirus B19 in kidney transplant recipients - a prospective study. Transpl Int.

[10] Locke JE, Zachary AA, Warren DS, Segev DL, Houp JA, Montgomery RA, Leffell MS (2009). Proinflammatory events are associated with significant increases in breadth and strength of HLA-specific antibody. Am J Transplant.

[11] Zachary AA, Montgomery RA, Locke JE, Leffell MS (2010). Proinflammatory events and HLA antibodies. Am J Transplant.

[12] Moudgil A, Nast CC, Bagga A (2001). Association of parvovirus B19 infection with idiopathic collapsing glomerulopathy. Kidney Int.

[13] Komatsuda A, Ohtani H, Nimura T, Yamaguchi A, Wakui H, Imai H, Miura AB (2000). Endocapillary proliferative glomerulonephritis in a patient with parvovirus B19 infection. Am J Kidney Dis.

